# Inspiratory muscle strength and skeletal muscle mass are associated with postoperative functional exercise capacity after lung cancer surgery: A retrospective cohort study

**DOI:** 10.1097/MD.0000000000051190

**Published:** 2026-10-23

**Authors:** Hanee Rim, Soo Ho Lee, Joon-Sung Kim, Bo Young Hong, Yeun Jie Yoo, Sun Im, Mi-Jeong Yoon

**Affiliations:** a Department of Rehabilitation Medicine, St. Vincent’s Hospital, College of Medicine, The Catholic University of Korea, Seoul, Republic of Korea; b Department of Rehabilitation Medicine, Bucheon St. Mary’s Hospital, College of Medicine, The Catholic University of Korea, Seoul, Republic of Korea.

**Keywords:** functional exercise capacity, incremental shuttle walking distance, inspiratory muscle strength, lung cancer, sarcopenia, skeletal muscle mass

## Abstract

Although surgical resection is the cornerstone of curative treatment for early-stage lung cancer, patients often experience postoperative declines in physical fitness and skeletal muscle mass, resulting in reduced exercise capacity, which is associated with prognosis. This study aimed to identify clinical factors associated with postoperative functional exercise capacity, as measured by the incremental shuttle walk test, in patients undergoing lung cancer surgery. This retrospective study included 62 subjects (mean age, 67 ± 8.7 years; 24 women and 38 men) who underwent lung cancer surgery and completed a postoperative assessment at 3 months. Functional exercise capacity was assessed using the incremental shuttle walking distance (ISWD) as the primary outcome. Correlation and regression analyses were conducted to examine the associations between ISWD and concurrent measures of forced expiratory volume in 1 second, forced vital capacity, maximal inspiratory pressure (MIP), maximal expiratory pressure, and skeletal muscle index (SMI). At 3 months postoperatively, ISWD was negatively correlated with age (*r* = −0.354, *P* = .005) and positively correlated with SMI (*r* = 0.253, *P* = .048) and predicted MIP (%; *r* = 0.293, *P* = .02), whereas other pulmonary function parameters were not significantly associated with ISWD. In regression models adjusted for 1-month ISWD, age, and sex, both predicted MIP (%; β = 0.290, *P* = .03; Δ*R*^2^ = 0.079) and SMI (β = 0.340, *P* = .03; Δ*R*^2^ = 0.075) were independently associated with 3-month ISWD. Subjects with low SMI demonstrated significantly lower 3-month ISWD than those with normal SMI (*P* = .02). Skeletal muscle mass and inspiratory muscle strength were associated with functional exercise capacity assessed using the incremental shuttle walk test at 3 months after lung cancer surgery. These findings suggest that assessment of muscle-related factors may improve the evaluation of postoperative functional recovery and help identify potential targets for rehabilitation.

## 1. Introduction

Lung cancer remains the leading cause of cancer-related mortality worldwide. Surgical resection is the cornerstone of curative treatment for early- and locally advanced-stage non-small cell lung cancer. However, lung resection imposes substantial physiological stress on both the respiratory and musculoskeletal systems, often leading to a significant decline in exercise capacity.^[1]^ Exercise capacity may decrease by up to 20% in the early postoperative period.^[2,3]^ Although recovery generally occurs gradually and reaches a plateau approximately 3 months after surgery, a subset of patients fails to regain their preoperative functional status.^[4,5]^ This reduction is clinically meaningful, as it has been associated with decreased physical activity, impaired quality of life, and poorer survival outcomes.^[6]^ The incremental shuttle walk test (ISWT) – a standardized, externally paced field test in which individuals walk progressively faster around a 10-m course until exhaustion – is widely used to assess functional exercise capacity in this population.^[7]^ Therefore, evaluating functional exercise capacity and its associated clinical factors during the recovery period is essential for guiding clinical management and appropriate intervention.

Traditionally, pulmonary function indices such as forced expiratory volume in 1 second (FEV_1_) have been widely used to assess preoperative risk and postoperative functional status, and the decline in exercise capacity after surgery has been attributed primarily to the loss of pulmonary function caused by lung parenchymal resection.^[8–10]^ However, the relationship between conventional spirometric indices – such as forced vital capacity (FVC) and FEV_1_ – and walking-test performance has been inconsistent across studies, with several reports demonstrating only weak or nonsignificant correlations.^[9,11]^ This discrepancy suggests that mechanisms beyond pulmonary mechanical impairment, such as respiratory muscle weakness and loss of peripheral skeletal muscle mass, may contribute substantially to the exercise limitation observed after thoracic surgery.

Respiratory muscle strength, commonly assessed by maximal inspiratory pressure (MIP) and maximal expiratory pressure (MEP), declines significantly after thoracic surgery and has been associated with functional exercise capacity in the early postoperative period.^[12,13]^ Separately, loss of skeletal muscle mass, or sarcopenia, is a well-recognized determinant of physical performance and exercise tolerance in patients with lung cancer, particularly in the preoperative and advanced-disease settings.^[14,15]^ Despite these individual lines of evidence, few studies have simultaneously examined how respiratory muscle strength, skeletal muscle mass, and conventional pulmonary function indices relate to exercise capacity at an intermediate postoperative time point. The third postoperative month represents a clinically meaningful window: the acute surgical recovery phase has generally resolved and pulmonary function has largely stabilized, yet patients are actively returning to daily life, making this an appropriate time to identify modifiable targets for rehabilitation. Whether functional exercise capacity at this stage is more closely related to respiratory muscle strength, skeletal muscle mass, or pulmonary function itself, however, remains insufficiently determined in the postoperative lung cancer population.

Therefore, in this retrospective analysis, we evaluated the associations of respiratory muscle strength, skeletal muscle index (SMI), and pulmonary function with functional exercise capacity assessed by ISWT at 3 months after lung cancer surgery.

## 2. Methods

### 2.1. Study design and subjects

This retrospective observational cohort study was conducted at a single hospital. At our institution, all patients undergoing lung cancer resection are routinely referred for postoperative rehabilitation evaluation as part of standard clinical practice; therefore, no additional selection based on clinical indication was applied. We reviewed consecutive patients who underwent lung cancer resection and were referred for postoperative rehabilitation evaluation between January 2024 and December 2025.

Of the 86 adult subjects who underwent surgical resection during the study period, 24 were excluded because of missing 3-month follow-up data for SMI, pulmonary function indices (FVC, FEV_1_), respiratory muscle strength (MIP, MEP), or incremental shuttle walking distance (ISWD). Of these, 16 subjects were lost to follow-up for reasons unrelated to their clinical condition (e.g., failure to attend the scheduled visit), and 8 subjects did not undergo follow-up evaluation because of clinical deterioration, including postoperative complications. Ultimately, 62 subjects with complete evaluations were included in the final analysis. Baseline characteristics collected included age, sex, history of cardiovascular disease, chronic obstructive pulmonary disease (COPD), type of surgery, and receipt of neoadjuvant chemotherapy.

### 2.2. Measurements

SMI (kg/m^2^) was obtained using multifrequency bioelectrical impedance analysis (InBody S10, InBody Co., Ltd., Seoul, Republic of Korea) and calculated as appendicular skeletal muscle mass divided by height squared. Body mass index (BMI) was calculated as body weight in kilograms divided by height in meters squared (kg/m^2^), and obesity was defined as BMI ≥ 25 kg/m^2^ according to the criteria of the Korean Society for the Study of Obesity.^[16]^ Pulmonary function was assessed by FVC and FEV_1_ and expressed as a percentage of the predicted value (%). Respiratory muscle strength was evaluated by MIP and MEP using a digital spirometer (Pony FX, COSMED, Rome, Italy) and expressed both as absolute values (cm H_2_O) and percentages of the predicted values (%). Functional exercise capacity was assessed using ISWT, an externally paced field test in which subjects walk at a progressively increasing pace until symptom-limited exhaustion or failure to maintain the required pace.

The variables evaluated in the present analyses were measured at 3 months after surgery, including ISWD, SMI, pulmonary function (FVC and FEV_1_), and respiratory muscle strength (MIP and MEP). In addition, ISWD was also assessed at 1 month after surgery.

### 2.3. Statistical analysis

Continuous variables were expressed as means with standard deviations if normally distributed, or medians with interquartile ranges if not. Categorical variables were presented as counts and percentages. Normality was assessed using the Shapiro–Wilk test and visual inspection of Q–Q plots. Pearson’s correlation coefficients were used for primary correlation analyses. Spearman correlation coefficients were additionally calculated as sensitivity analyses to assess the robustness of the findings. All statistical tests were two-tailed.

Correlation and univariable linear regression analyses were performed to evaluate associations between 3-month ISWD and each candidate variable (SMI, MIP, MEP, FEV_1_, and FVC). Multivariable linear regression models were constructed to examine independent associations with 3-month ISWD, adjusting for 1-month ISWD, age, and sex as baseline covariates. Age and sex were retained as demographic variables. The ISWD at 1 month was included to account for interindividual differences in early postoperative functional status and functional exercise capacity. Other candidate variables were considered for inclusion when they showed a statistically significant association with 3-month ISWD in the univariable analyses (*P* < .05). For each variable of interest, Δ*R*^2^ was calculated as the increase in *R*^2^ when the variable was added to the baseline model (1-month ISWD + age + sex), and the significance of this improvement was assessed using an *F*-change test. Multicollinearity was assessed using variance inflation factors (VIFs). Given the relatively limited sample size, internal validation was subsequently performed using 2000 bootstrap resamples and leave-one-out cross-validation (LOOCV) to assess model stability and potential optimism.

To evaluate potential differences in postoperative functional exercise capacity by surgical extent and approach, 3-month ISWD was additionally compared between groups. Surgical procedures were categorized by extent of pulmonary resection into major resection (lobectomy- or pneumonectomy-based procedures, including combined resections involving lobectomy) and limited resection (segmentectomy- or wedge-based procedures without lobectomy). Surgical approach was categorized as video-assisted thoracoscopic surgery (VATS) or robotic surgery. Three-month ISWD was also compared between participants with and without obesity (BMI ≥ 25 kg/m^2^). Group comparisons were performed using the independent-samples *t* test or Mann–Whitney *U* test, as appropriate based on the distribution of the data. As exploratory analyses, mediation analysis was performed to examine whether the association between SMI and 3-month ISWD was mediated by inspiratory muscle strength. Indirect effects were estimated using a regression-based mediation model with bias-corrected bootstrap confidence intervals (CIs) generated from 5000 resamples. Statistical significance of the indirect effect was determined based on whether the 95% CI excluded 0.

Subjects were also classified as sarcopenic or non-sarcopenic according to the Asian Working Group for Sarcopenia (AWGS) 2019 criteria,^[17]^ using SMI, handgrip strength, and gait speed criteria, and 3-month ISWD was compared between low- and normal-SMI groups using the Mann–Whitney *U* test. Additional subgroup comparisons were performed between sarcopenic and non-sarcopenic SMI groups. A two-tailed *P* value < .05 was considered statistically significant. All statistical analyses were performed using Jamovi (version 2.7.18.0; The Jamovi Project, Sydney, Australia), Python (version 3.13; Python Software Foundation), with the statsmodels (version 0.14; statsmodels developers, open-source project sponsored by NumFOCUS, Austin) and NumPy (version 2.4; NumPy Developers, open-source project sponsored by NumFOCUS, Austin) packages.

## 3. Results

### 3.1. Participant characteristics

The mean age was 67.0 ± 8.7 years, and 38 subjects (61.3%) were male. Cardiovascular disease was present in 3 subjects (4.8%), and COPD in 14 subjects (22.6%). Neoadjuvant chemotherapy had been administered to 9 subjects (14.5%). Regarding surgical procedures, lobectomy was the most common (n = 33, 53.2%), followed by segmentectomy (n = 11, 17.7%), wedge resection (n = 5, 8.1%), and pneumonectomy (n = 1, 1.6%). Combined resections included lobectomy with wedge resection (n = 8, 12.9%), segmentectomy with wedge resection (n = 3, 4.8%), and lobectomy with segmentectomy + wedge resection (n = 1, 1.6%). Regarding the surgical approach, 50 patients (80.6%) underwent VATS and 12 (19.4%) underwent robotic surgery; none underwent open thoracotomy. At 3 months after surgery, the mean FVC was 2.65 ± 0.75 L (90.4% ± 20.2% predicted), and the mean FEV_1_ was 1.96 ± 0.57 L (84.0% ± 19.3% predicted). The mean MIP and MEP were 90.15 ± 28.82 cm H_2_O and 80.94 ± 24.78 cm H_2_O, respectively. The mean ISWD was 370.3 ± 131.2 m, and the mean SMI was 7.3 ± 1.2 kg/m^2^. Obesity, defined as BMI ≥ 25 kg/m^2^, was present in 19 patients (30.6%; Table 1).

**Table 1:** Demographic and clinical characteristics of the study participants.

Variables	Values
Subjects, n	62
Age, yr	67.0 ± 8.7
Male sex, n (%)	38 (61.3)
Comorbidity	
Cardiovascular disease, n (%)	3 (4.8)
COPD, n (%)	14 (22.6)
Neoadjuvant chemotherapy, n (%)	9 (14.5)
Sarcopenia, n (%)	9 (14.5)
Obesity, n (%)	19 (30.6)
BMI (kg/m^2^)	23.9 (3.79)
Surgical procedure, n (%)	
Major	43 (69.4)
Lobectomy	33 (53.2)
Pneumonectomy	1 (1.6)
Lobectomy + wedge resection	8 (12.9)
Lobectomy + segmentectomy + wedge resection	1 (1.6)
Limited	19 (30.6)
Segmentectomy	11 (17.7)
Wedge resection	5 (8.1)
Segmentectomy + wedge resection	3 (4.8)
Surgical approach, n (%)	
VATS (video-assisted thoracoscopic surgery)	50 (80.6)
Robotic surgery	12 (19.4)
Postoperative variables (3 mo after surgery)	
FEV_1_ (%)	84.0 ± 19.3
FEV_1_ (L)	1.89 (0.85)
FVC (%)	90.5 (24.5)
FVC (L)	2.65 ± 0.75
MIP (cm H_2_O)	90.15 ± 28.82
MIP (%)	109.1 ± 33.3
MEP (cm H_2_O)	80.94 ± 24.78
MEP (%)	79.0 (31.0)
ISWD (m)	370.3 ± 131.2
SMI (kg/m^2^)	7.3 ± 1.2

Values are presented as number (%), mean ± standard deviation, or median (interquartile range), as appropriate based on the data distribution assessed using the Shapiro–Wilk test. Major resection included lobectomy- or pneumonectomy-based procedures, including combined resections involving lobectomy. Limited resection included segmentectomy- or wedge-based procedures without lobectomy. Obesity was defined as BMI ≥ 25 kg/m^2^ according to the criteria of the Korean Society for the Study of Obesity.

BMI = body mass index, COPD = chronic obstructive pulmonary disease, FEV_1_ = forced expiratory volume in 1 second, FVC = forced vital capacity, ISWD = incremental shuttle walking distance, MEP = maximal expiratory pressure, MIP = maximal inspiratory pressure, SMI = skeletal muscle index.

In exploratory analyses, surgical procedures were categorized according to the extent of pulmonary resection into major resection (lobectomy- or pneumonectomy-based) and limited resection (segmentectomy- or wedge-based). No significant difference in 3-month ISWD was observed between the major resection (n = 43) and limited resection (n = 19) groups (358 ± 133 vs 398 ± 127 m, *P* = .27). The median 3-month ISWD was 350 (285–440) m in the VATS group and 445 (410–463) m in the robotic surgery group (*P* = .050). Similarly, 3-month ISWD did not differ significantly between nonobese and obese patients (366 ± 137 m vs 381 ± 120 m, respectively; *P* = .672).

### 3.2. Univariable correlations with postoperative functional exercise capacity

Correlation analyses were performed to evaluate associations between postoperative functional exercise capacity, assessed using 3-month ISWD, and clinical variables (Table 2). Age was negatively correlated with 3-month ISWD (*r* = −0.354, *P* = .005). Pulmonary function measured at 3 months after surgery demonstrated no significant correlations with 3-month ISWD: predicted FEV_1_ (%) and FVC (%) were positively but nonsignificantly correlated with ISWD (FEV_1_ [%]: *r* = 0.239, *P* = .061; FVC [%]: *r* = 0.186, *P* = .15).

**Table 2:** Correlations between 3-month incremental shuttle walking distance and clinical variables.

Variables	*r* *	*P* value
	ISWD	
Age (yr)	−0.354	.005†
Sex	0.137	.29
1-mo ISWD	0.453	.001†
MIP (%)	0.293	.021†
MIP (cm H_2_O)	0.229	.074
MEP (%)	0.147	.26
MEP (cm H_2_O)	0.087	.50
FEV_1_ (%)	0.239	.061
FEV_1_ (L)	0.242	.058
FVC (%)	0.186	.15
FVC (L)	0.191	.14
SMI (kg/m^2^)	0.253	.048†

	MIP (%)	
SMI (kg/m^2^)	0.281	.027†

FEV_1_ = forced expiratory volume in 1 second, FVC = forced vital capacity, ISWD = incremental shuttle walking distance, MEP = maximal expiratory pressure, MIP = maximal inspiratory pressure, SMI = skeletal muscle index.

* Data are presented as Pearson correlation coefficients (*r*).

† Statistical significance was defined as *P* < .05.

Among respiratory muscle strength measures, predicted MIP (%) was positively correlated with ISWD (*r* = 0.293, *P* = .021), whereas absolute MIP (cm H_2_O) showed a borderline association (*r* = 0.229, *P* = .074). Neither predicted nor absolute MEP was significantly associated with ISWD (MEP [%]: *r* = 0.147, *P* = .26; MEP [cm H_2_O]: *r* = 0.087, *P* = .50). SMI at 3 months was positively correlated with 3-month ISWD (*r* = 0.253, *P* = .048). In addition, SMI was significantly correlated with predicted MIP (%; *r* = 0.281, *P* = .027) and MIP (cm H_2_O; *r* = 0.527, *P* < .001).

### 3.3. Multivariable correlations with postoperative functional exercise capacity

Multivariable linear regression analyses were performed to identify independent factors associated with 3-month ISWD (Table 3). The baseline model, including 1-month ISWD, age, and sex, explained 30.5% of the variance in 3-month ISWD (*R*^2^ = 0.305).

**Table 3:** Multivariable linear regression analyses for 3-month postoperative incremental shuttle walking distance.

Variables	Baseline + MIP		Baseline + SMI		Baseline + MIP + SMI	
	β	*P* value	β	*P* value	β	*P* value
Age	−0.309	**.017** *	−0.226	.089	−0.250	.059
Sex (male)	−0.008	.97	0.505	.094	0.295	.36
1-mo ISWD	0.372	**.005** *	0.335	**.011** *	0.340	**.009** *
3-mo MIP (%)	0.290	**.026** *	–	–	0.216	.11
3-mo SMI (kg/m^2^)	–	–	0.340	**.029** *	0.246	.12
Total *R*^2^	0.384		0.380		0.418	
Adjusted *R*^2^	0.325		0.321		0.347	
*F (P* value)	6.54 (<.001)		6.44 (<.001)		5.89 (<.001)	
Bootstrap-corrected *R*^2^	0.174		0.182		0.179	
LOOCV *R*^2^ (*Q*^2^)	0.170		0.179		0.176	

Data are presented as standardized regression coefficients (β) and corresponding *P* values. Baseline-adjusted models included age, sex, and 1-month postoperative ISWD as covariates. Total *R*^2^ and adjusted *R*^2^ indicate the proportion of variance in 3-month ISWD explained by each model. The *F* statistic represents the overall significance of each regression model. Bootstrap-corrected *R*^2^ was calculated as the apparent *R*^2^ minus the mean optimism estimated from 2000 bootstrap resamples. *Q*^2^ represents the cross-validated *R*^2^ obtained using leave-one-out cross-validation.

ISWD = incremental shuttle walking distance, LOOCV = leave-one-out cross-validation, MIP = maximal inspiratory pressure, SMI = skeletal muscle index.

* Statistical significance was defined as *P* < .05.

When predicted MIP (%) was added to the baseline model, it was independently associated with 3-month ISWD (β = 0.290, *P* = .026), increasing the model *R*^2^ to 0.384 (Δ*R*^2^ = 0.079). Similarly, when SMI was added to the baseline model, it was independently associated with 3-month ISWD (β = 0.340, *P* = .029), increasing the model *R*^2^ to 0.380 (Δ*R*^2^ = 0.075).

When MIP (%) and SMI were simultaneously included, the model *R*^2^ further increased to 0.418 (Δ*R*^2^ = 0.113 vs the baseline model); however, neither MIP (%) nor SMI remained independently significant (β = 0.216, *P* = .11 and β = 0.246, *P* = .12, respectively). VIFs were low in this combined model (1.23 for predicted MIP and 1.77 for SMI; see Supplemental Table 1, Supplemental Digital Content 1), indicating no problematic multicollinearity. Furthermore, a supplementary mediation analysis did not demonstrate a significant indirect effect of SMI on 3-month ISWD through inspiratory muscle strength (indirect effect estimate = 1.06, 95% CI: −0.08 to 3.27, *P* = .14).

To assess potential optimism and stability of the regression models given the limited sample size, internal validation was performed using 2000 bootstrap resamples and LOOCV. The apparent *R*^2^ values of 0.380 to 0.418 decreased to 0.174 to 0.182 after bootstrap correction, while the LOOCV *R*^2^ values ranged from 0.170 to 0.179, indicating some degree of optimism in the apparent model performance (Table 3). In bootstrap analyses, 1-month ISWD remained significant in 85% to 91% of samples, followed by age (57%–81%), 3-month MIP (65.4%), and 3-month SMI (63.5%; Supplemental Table 2, Supplemental Digital Content 2).

### 3.4. Comparison of 3-month functional exercise capacity according to SMI status

In a supplementary subgroup analysis, postoperative 3-month ISWD was compared according to skeletal muscle mass status, classified using AWGS 2019 sex-specific SMI cutoff values. Fourteen subjects were classified as having low SMI, whereas 48 subjects were classified as having normal SMI. Subjects in the low-SMI group had significantly lower 3-month ISWD than those in the normal-SMI group (Mann–Whitney *U* test, *P* = .02; Fig. 1).

**Figure 1.**
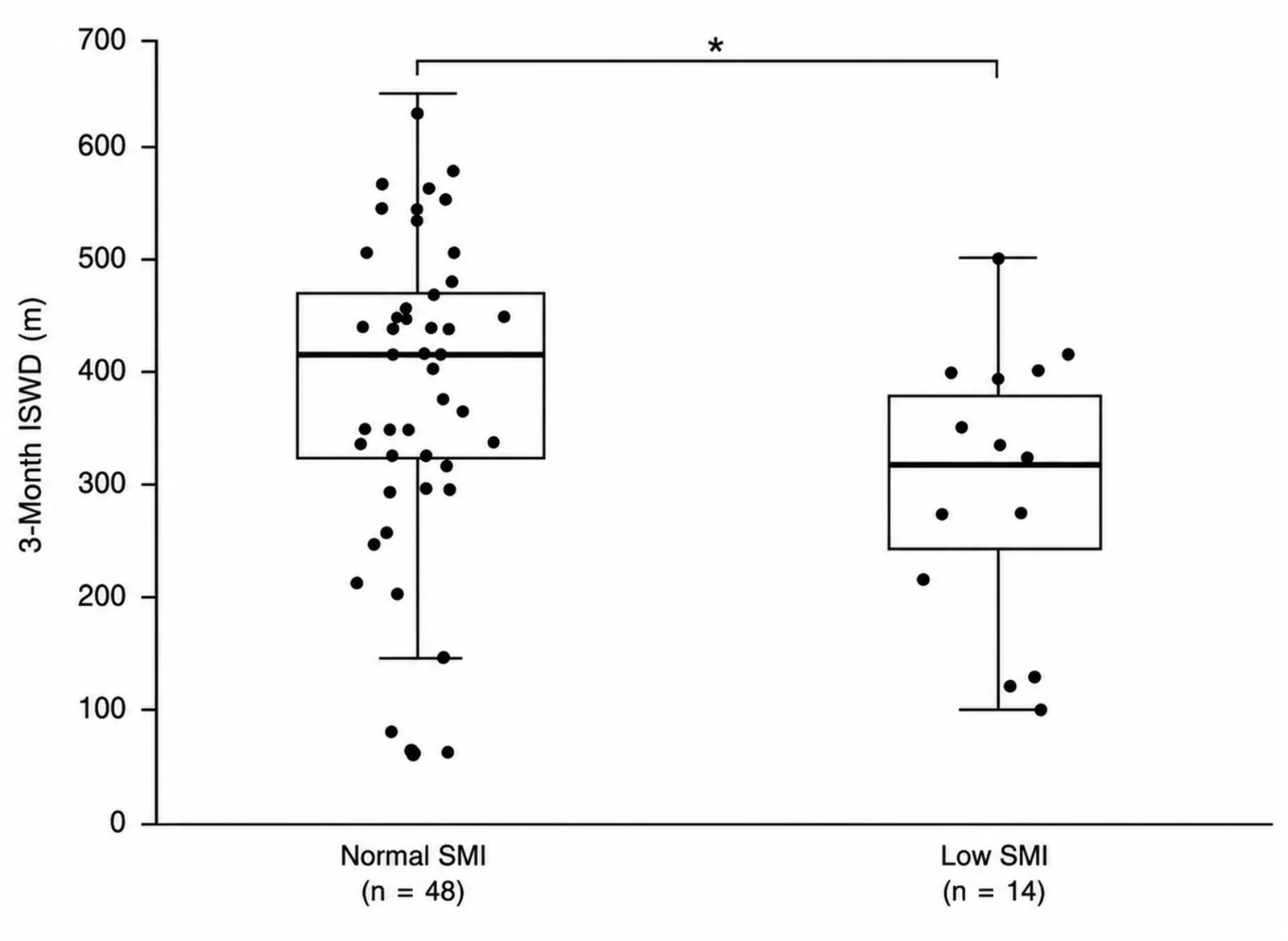
Comparison of 3-month postoperative incremental shuttle walking distance according to SMI status. Three-month ISWD was compared between subjects with low and normal SMI based on the Asian Working Group for Sarcopenia (AWGS) 2019 sex-specific cutoff values (male < 7.0 kg/m^2^ and female < 5.7 kg/m^2^). Subjects with low SMI demonstrated significantly lower ISWD than those with normal SMI. Box plots show the median, 25th percentile, and 75th percentile. Whiskers represent the minimum and maximum observed values. Individual dots represent individual subjects. *P = .02. ISWD = incremental shuttle walking distance, SMI = skeletal muscle index.

As an additional exploratory analysis, subjects were further classified according to the AWGS 2019 sarcopenia criteria (incorporating SMI, handgrip strength, and gait speed). Among the 57 subjects with complete data for this classification, 9 met the diagnostic criteria for sarcopenia. Consistent with the SMI-based comparison, the sarcopenia group showed significantly lower 3-month ISWD than the non-sarcopenia group (Student’s *t* test, *P* = .038; Mann–Whitney *U* test, *P* = .035; Supplemental Figure 1, Supplemental Digital Content 3).

## 4. Discussion

The main findings of this study were that both skeletal muscle mass and inspiratory muscle strength were associated with postoperative functional exercise capacity evaluated by ISWT at 3 months after lung cancer surgery, whereas statistically significant associations between spirometric lung function – represented by FEV_1_ and FVC – and 3-month ISWD were not observed. In separate adjusted models, both SMI and predicted MIP (%) remained significantly associated with 3-month ISWD after adjustment for 1-month ISWD, age, and sex. However, when both variables were included simultaneously, neither remained individually significant, despite a further increase in the overall explanatory power of the model. These findings suggest that skeletal muscle mass and inspiratory muscle strength may capture partially overlapping but complementary information regarding functional exercise capacity at 3 months after lung cancer surgery.

Respiratory muscle strength, particularly inspiratory muscle strength, emerged as a significant determinant of functional exercise capacity in this study. MIP reflects the strength of the diaphragm and accessory inspiratory muscles, which are essential for maintaining adequate ventilation during physical activity. Inspiratory muscle weakness increases the work of breathing and may contribute to earlier onset of ventilatory limitation during exercise, thereby limiting exercise tolerance.^[18]^ This is consistent with previous studies showing that postoperative inspiratory muscle training improved both respiratory muscle function and exercise capacity.^[19,20]^ Improvements in MIP are accompanied by enhancements in peak oxygen uptake and functional exercise performance, suggesting a physiologically meaningful link between respiratory muscle function and overall exercise capacity.^[19,21,22]^ Our findings support the potential clinical relevance of assessing respiratory muscle strength in the postoperative setting.

In addition to respiratory muscle strength, skeletal muscle mass was associated with functional exercise capacity evaluated by ISWT. Skeletal muscle plays a central role in oxygen utilization during exercise, and reductions in muscle mass may impair aerobic metabolism and functional performance. In our supplementary analyses, subjects with low SMI demonstrated significantly lower 3-month ISWD than those with normal SMI. Similar findings were observed when the analysis was extended to sarcopenia defined according to the AWGS 2019 criteria. These findings are in line with previous reporting that sarcopenia is associated with impaired physical function, reduced physical activity, and poorer postoperative outcomes in patients with lung cancer.^[23–25]^ However, these subgroup findings should be interpreted with caution given the limited number of subjects with sarcopenia in our cohort.

Interestingly, SMI and MIP were significantly correlated with each other, suggesting a potential shared physiological basis between skeletal muscle status and respiratory muscle performance. This association has also been reported in other populations: studies in older adults and patients with chronic respiratory disease have similarly demonstrated associations between systemic skeletal muscle mass and respiratory muscle strength.^[26–28]^ These findings support the notion that respiratory muscle performance may, at least in part, reflect generalized skeletal muscle status rather than a purely localized phenomenon.

In our multivariable model, when SMI and MIP were included simultaneously, neither remained individually significant despite improvement in overall model performance. The VIF in this combined model was low (Supplemental Table 1, Supplemental Digital Content 1), suggesting that this attenuation was not driven by problematic multicollinearity but rather may reflect overlapping physiological domains shared between skeletal muscle mass and respiratory muscle performance. Furthermore, a supplementary mediation analysis did not demonstrate a significant indirect effect of SMI on 3-month ISWD through MIP. Taken together, these findings suggest that skeletal muscle mass and respiratory muscle performance may share common physiological mechanisms while still contributing distinct and complementary information regarding postoperative functional exercise capacity. A similar concept has been described in chronic respiratory disease, particularly COPD, where respiratory muscle dysfunction and peripheral skeletal muscle impairment coexist through shared systemic mechanisms, while also reflecting distinct local physiological adaptations.^[29]^ For example, respiratory muscles are strongly influenced by altered ventilatory mechanics and chronic respiratory loading, whereas peripheral skeletal muscles are more closely affected by physical inactivity and deconditioning.^[30,31]^ Therefore, evaluating both factors may provide a more comprehensive understanding of postoperative functional status.

In contrast, we did not observe statistically significant associations between FEV_1_ or FVC and 3-month ISWD in this study. This finding is in line with previous studies suggesting that spirometric parameters alone may not fully explain the variability in functional performance after lung resection.^[32,33]^ Moreover, improvements in exercise capacity have been observed at 3 months after surgery despite persistent impairment in FEV_1_, suggesting that factors other than pulmonary function may also contribute to functional recovery during this period.^[34]^ However, our relatively small sample size and limited statistical power may also have reduced our ability to detect modest associations between spirometric parameters and ISWD. Therefore, the absence of statistically significant associations should not be interpreted as evidence that pulmonary function is unrelated to postoperative functional exercise capacity. Rather, spirometric measures alone may not fully capture the multifactorial determinants of postoperative functional exercise capacity.

Our findings have potential clinical implications. Both inspiratory muscle strength and skeletal muscle mass may serve as clinically relevant targets for intervention and risk stratification after lung cancer surgery. Inspiratory muscle strength is a modifiable factor that can be improved through targeted interventions such as inspiratory muscle training, while skeletal muscle mass may be influenced by nutritional support and resistance exercise. Previous studies have demonstrated that pulmonary rehabilitation, incorporating resistance exercise and inspiratory muscle training, can improve respiratory muscle strength, exercise capacity, and postoperative recovery in patients undergoing lung cancer surgery.^[19,35,36]^ Building on these findings, our results suggest that pulmonary rehabilitation strategies addressing both respiratory muscle performance and systemic skeletal muscle status may be beneficial for improving postoperative exercise capacity in this population.

This study has several limitations. First, the relatively small sample size is an important limitation of this study. Given the number of patients and predictor variables, the regression estimates may be susceptible to sampling variability and overfitting. To address this concern, we performed bootstrap internal validation and LOOCV. The lower internally validated *R*^2^ values compared with the apparent *R*^2^ values suggest some optimism in the original model performance and support cautious interpretation of the regression results. Future studies involving larger cohorts and external validation are needed to determine whether these associations are reproducible and generalizable.

Second, among the subjects excluded from the analysis, some did not undergo follow-up evaluation because of clinical deterioration. This raises the possibility that our final cohort may be skewed toward subjects with relatively favorable postoperative recovery, potentially underestimating the true burden of exercise intolerance in the broader postoperative population.

Third, because SMI, respiratory muscle strength, pulmonary function, and ISWD were assessed concurrently at 3 months after surgery, the primary analyses are cross-sectional in nature. To account for differences in earlier postoperative exercise capacity, we additionally adjusted for 1-month ISWD in the multivariable models. However, this adjustment does not establish longitudinal or causal relationships between the physiological measures and postoperative functional exercise capacity, nor does it determine whether the observed associations are specifically attributable to surgery. Future prospective studies incorporating preoperative and serial postoperative assessments are needed to clarify the longitudinal relationships and contributions of these measures to functional recovery after lung cancer surgery.

Fourth, the ISWT represents a specific dimension of functional exercise capacity and may not capture all aspects of postoperative exercise capacity.^[37]^ Performance tests with different physiological demands may yield different relationships with pulmonary and muscular factors. For example, stair-climbing tests impose different cardiorespiratory and muscular demands from walking-based tests, and postoperative changes in stair-climbing performance have been associated with changes in pulmonary function, including FEV_1_.^[38]^ Complementary exercise assessments may therefore provide a more comprehensive characterization of postoperative functional exercise capacity.

Fifth, all patients in this cohort underwent minimally invasive surgery, and approximately one-third were classified as having obesity according to Korean criteria. Therefore, the generalizability of our findings to patients undergoing open thoracotomy or to populations with different body composition characteristics remains uncertain.

Finally, multiple correlation and regression analyses were performed without adjustment for multiple comparisons; therefore, the reported associations, particularly those with *P* values close to the .05 threshold, should be interpreted as exploratory and hypothesis-generating rather than confirmatory, and warrant validation in larger prospective cohorts. Relatedly, in the exploratory analysis, the number of subjects classified as having sarcopenia was small (n = 9), further limiting the statistical power of this subgroup comparison and warranting cautious interpretation. Skeletal muscle mass was assessed using bioelectrical impedance analysis, which may be influenced by hydration status and may not fully reflect muscle quality, muscle composition, or neuromuscular function.

## 5. Conclusion

Skeletal muscle mass and inspiratory muscle strength were associated with functional exercise capacity assessed by the ISWT at 3 months after lung cancer surgery. These findings highlight the potential value of assessing skeletal muscle status and respiratory muscle strength during postoperative recovery and suggest that these measures may help inform individualized rehabilitation planning in this population.

## Acknowledgments

The authors acknowledge using ChatGPT (GPT-5.5, OpenAI) for text editing and refining the presentation to improve fluency in English during the preparation of this manuscript on June 25, 2026. The authors affirm that the original intent and meaning of the content remain unaltered during editing and that ChatGPT had no involvement in shaping the intellectual content of this work. All scientific content, study design, data analysis, interpretation, and final decisions were developed, verified, and approved by the authors, who take full responsibility for the final manuscript.

## Author contributions

**Conceptualization:** Mi-Jeong Yoon.

**Data curation:** Hanee Rim, Soo Ho Lee.

**Formal analysis:** Hanee Rim, Soo Ho Lee.

**Funding acquisition:** Sun Im, Mi-Jeong Yoon.

**Investigation:** Joon-Sung Kim, Bo Young Hong, Yeun Jie Yoo, Sun Im, Mi-Jeong Yoon.

**Methodology:** Hanee Rim.

**Project administration:** Mi-Jeong Yoon.

**Resources:** Joon-Sung Kim, Bo Young Hong, Yeun Jie Yoo.

**Supervision:** Sun Im, Mi-Jeong Yoon.

**Validation:** Hanee Rim.

**Visualization:** Hanee Rim, Soo Ho Lee.

**Writing – original draft:** Hanee Rim.

**Writing – review & editing:** Soo Ho Lee, Joon-Sung Kim, Bo Young Hong, Yeun Jie Yoo, Mi-Jeong Yoon.






