# Supplementary material for: Inspiratory muscle strength and skeletal muscle mass are associated with postoperative functional exercise capacity after lung cancer surgery: A retrospective cohort study
Source: Medicine (Baltimore). 2026 Oct 23;105(43):e51190. doi: 10.1097/MD.0000000000051190 (PMC13706440; doi:10.1097/MD.0000000000051190)
Supplement: Supplementary file 1 [file medi-105-e51190-s001.docx]

**Supplemental Table 1. Variance Inflation Factors for Variables Included in the Combined Regression Model**

|  | **VIF** |
| --- | --- |
| Age | 1.16* |
| Sex | 1.76* |
| 1-month ISWD | 1.08* |
| 3-month MIP (%) | 1.23* |
| 3-month SMI (kg/m²) | 1.77* |

Values represent variance inflation factors (VIFs) from the combined multivariable regression model including age, sex, 1-month ISWD, 3-month MIP (% predicted), and 3-month SMI.

*VIF values < 5 indicate no evidence of problematic multicollinearity.

Abbreviations: VIF= variance inflation factor, ISWD=incremental shuttle walking distance, MIP= maximal inspiratory pressure, SMI= skeletal muscle index
