# Supplementary material for: Inspiratory muscle strength and skeletal muscle mass are associated with postoperative functional exercise capacity after lung cancer surgery: A retrospective cohort study
Source: Medicine (Baltimore). 2026 Oct 23;105(43):e51190. doi: 10.1097/MD.0000000000051190 (PMC13706440; doi:10.1097/MD.0000000000051190)
Supplement: Supplementary file 2 [file medi-105-e51190-s002.docx]

**Supplemental table 2. Bootstrap stability of regression coefficients across the three multivariable regression models**

| **Variable** | **Baseline**  **+ MIP + SMI**  **(%)** | **Baseline**  **+ MIP**  **(%)** | **Baseline**  **+ SMI**  **(%)** |
| --- | --- | --- | --- |
| Age | 59.5 | 81.4 | 57.2 |
| Sex | 21.2 | 11.2 | 34.5 |
| 1-month ISWD | 88.5 | 90.7 | 85.4 |
| 3-month MIP (%) | 41.8 | 65.4 | - |
| 3-month SMI (kg/m²) | 41.9 | - | 63.5 |

Values represent the percentage of 2,000 bootstrap resamples in which the predictor remained statistically significant (*P* < 0.05), with a consistent direction of effect in all cases. A dash indicates the variable was not included in that model.
